# The Effects of Supplementary Cr3 (Chromium(III) Propionate Complex) on the Mineral Status in Healthy Female Rats

**DOI:** 10.1007/s12011-017-0985-3

**Published:** 2017-03-10

**Authors:** Halina Staniek, Zbigniew Krejpcio

**Affiliations:** Department of Human Nutrition and Hygiene, Poznań University of Life Sciences, ul. Wojska Polskiego 31, 60-624 Poznań, Poland

**Keywords:** Chromium(III) propionate, Rats, Mineral status

## Abstract

More and more people use food supplements for various reasons, e.g. to prevent mineral deficiency and diseases (e.g. osteoporosis, diabetes, anaemia). Supplements containing Cr(III) are purchased primarily for weight loss and antidiabetic effects. The aim of this study was to evaluate the effects of supplementary Cr3 **{**chromium(III) propionate complex, [Cr_3_O(O_2_CCH_2_CH_3_)_6_(H_2_O)_3_]NO_3_)} on the mineral status in female Wistar rats. The study was carried out on 30 female Wistar rats, divided into five groups (six animals in each): a control group and test groups fed Cr3 supplemented diets with 100, 200, 500 and 1000 mg Cr · kg^−1^ diet (equivalent to 10, 20, 50 and 100 mg Cr ·kg^−1^ body mass (b.m.) per day) given as Cr3 for 4 weeks. Supplementary Cr3 increased the Cr content in tissues in a dose-dependent manner. High dietary doses of Cr3, 20 and 100 mg Cr · kg^−1^ b.m., increased the Cu content in the liver and spleen as well as the Zn content in the kidneys but decreased the liver Ca content. Doses of 50–100 mg Cr ·kg^−1^ b.m. decreased the serum Fe concentration and the Fe content in the liver and kidneys. Supplementation with Cr3 at doses of 10 and 100 mg Cr ·kg^−1^ b.m. did not affect the Mg content in the rats’ tissues. In conclusion, high dietary doses of Cr3 (10 and 100 mg Cr· kg^−1^ b.m.) given for 4 weeks affected the mineral status of Fe, Zn, Cu and Ca in the tissues of healthy female Wistar rats.

## Introduction

In recent years, chromium has been one of the most investigated dietary minerals. Chromium is a trace mineral that has received much attention as a dietary supplement because good dietary sources of chromium are scarce and the intake is usually low. Chromium(III) deficiency may contribute to carbohydrate metabolism disorder [1].

Many trials proved the positive effect of supplementary chromium(III) on fasting plasma glucose, lipid variables, especially in diabetic subjects [2–4]. For this reason, trivalent chromium has been postulated to be necessary for insulin efficacy in regulating the metabolism of carbohydrates, lipids and protein [5]. A number of chromium compounds can be considered as a perspective for metabolic syndrome treatment [6].

For over 50 years, chromium has generally been believed to be an essential trace element. However, the mechanism(s) of Cr action at the molecular level for this role and its essentiality have not been substantiated. Recent research has not supported the role of chromium [7].

In 2002, the Food and Nutrition Board of the US National Academy of Science set the Adequate Intake (AI) of chromium at 25 μg ·day^−1^ for adult women and 35 μg ·day^−1^ for men [8], which was lower than the previous recommended dietary intake of 50–200 μg per day. Recently, the EFSA panel found no evidence of beneficial effects associated with chromium intake in healthy subjects and concluded that setting the AI for chromium was not appropriate [5].

In general, the oral intake of chromium has low toxicity partially due to its poor absorption (about 0.5–2.0%). However, different Cr(III) compounds have diverse rates of absorption [9]. Organic Cr(III) forms have greater bioavailability than inorganic ones. It is well known that the mineral intake at high doses has antagonistic effects on other elements [9].

Chromium is one of the best-selling mineral supplements in the USA [10]. Trivalent chromium, the form found in food and dietary supplements, is considered to be safe. Many organic chromium complexes, including chromium picolinate [Cr(Pic)_3_], chromium nicotinate (NCB) [11, 12], chromium histidinate (CrHis) [13, 14], chromium complex of d-phenylalanine [Cr(d-Phe)_3_] [15, 16], chromium propionate complex (Cr3) [4, 17–19] and chromium glycinate complex (CrGly) [19], have been synthesised and demonstrated to be biologically effective. Different coordinate ligands of these organic chromium complexes exhibited different bioactive compounds [20].

For humans, a typical Cr intake is 20–45 μg per day in the diet [21], while doses of supplements may contain 200–1000 μg Cr(III) [1, 22]. These doses correspond to daily body weight-adjusted doses of 0.29–0.64 μg Cr(III)· kg^−1^ body mass (in the diet) and 2.86–14.3 μg Cr(III) ·kg^−1^ b.m. (in supplements) in an individual with an average weight of 70 kg [23].

Few studies have been designed to evaluate the effects of trivalent Cr supplementation on the content of Cr and other minerals in animal tissues. Chromium is distributed to various tissues of the body but appears to be most concentrated in the kidneys, liver and muscles [24]. Dietary Cr supplementation at high doses can potentially affect the mineral status due to possible interactions with other macro- and microelements at absorption, transport, metabolism, excretion and other levels [25]. In this case, the high supply of Cr(III) can affect the metabolism of other minerals in healthy rats. Therefore, the aim of this study was to evaluate the effects of high doses of chromium (III) complex with propionic acid, so-called Cr3 (100–1000 mg Cr ·kg^−1^ diet, equivalent to 10, 20, 50 and 100 mg Cr/kg b.m. per day) on the mineral status in healthy female rats.

## Material and Methods

### Test Chemicals

The chromium(III) complex with propionic acid in the form of nitrate salt (chemical formula [Cr_3_O(O_2_CCH_2_CH_3_)_6_(H_2_O)_3_]NO_3_ (Cr3) was synthesised in a laboratory at the Department of Product Ecology, Poznań University of Economics, Poland, according to the method described by Earnshaw et al. [26]. The Cr3 was found to contain 21% of elemental Cr, determined by the AAS method (spectrometer AAS-3 with BC correction, Zeiss, Germany).

### Animals and Diets

Thirty 10-week-old female Wistar rats were obtained from the Department of Toxicology, Poznań University of Medical Sciences, Poland. The animals were housed in single cages, at controlled temperature, photoperiod and air humidity (19–22 °C, 12-h light/dark cycle, 55–60% of ambient air humidity). After 5-day adaptation to laboratory conditions, the rats were divided into five equal groups (the control group and groups treated with Cr3—six animals in each group, equal body weight of 180 g). All the groups were fed a commercial diet for maintenance of adult rodents (*Labofeed H*), enriched with 0, 100, 200, 500 and 1000 mg Cr(III)/kg of diet (ca. 0, 10, 20, 50 and 100 mg Cr/kg b.m. per day) given as [Cr_3_O(O_2_CCH_2_CH_3_)_6_(H_2_O)_3_]NO_3_ for 4 weeks. Table 1 shows the composition of the basic *Labofeed H* diet. The Cr content in the basic diet (the control group) was 0.5 ± 0.06 mg ·kg^−1^, while in the supplemented diets it was 107.5 ± 6.5 mg· kg^−1^ (A); 224.8 ± 32.4 mg· kg^−1^ (B); 535.5 ± 26.22 mg· kg^−1^ (C) and 1049.5 ± 17.6 mg· kg^−1^ (D), respectively. The diets were stored at 4 °C. The rats were allowed free access to feed and distilled water throughout the whole experiment.

**Table 1:** The composition of basic *Labofeed H* diet in the experiment (mean ± SD)

Component	Unit	Content
Energy	MJ· 100 g^−1^	1.69 ± 0.03
Fat	%	3.16 ± 0.07
Protein	%	24.10 ± 0.21
Carbohydrates	%	54.96
Dry mass	%	88.73 ± 0.05
Ash	%	6.51 ± 0.11
Ca	g· kg^−1^	13.41 ± 1.61
Mg	g· kg^−1^	2.24 ± 0.06
Fe	mg· kg^−1^	239.49 ± 46.34
Zn	mg· kg^−1^	133.19 ± 42.31
Cu	mg· kg^−1^	20.42 ± 2.91

The feed intake was measured daily, while body weight gains were monitored weekly. At the end of the experiment, after 12-h starvation, the rats were euthanised by intraperitoneal injection of thiopental (40 mg· kg^−1^ body mass). Blood was collected into tubes; tissue samples (liver, kidneys, heart, spleen, pancreas, ovaries) were collected, weighed and frozen. The experimental protocol was approved by the Local Bioethical Commission in Poznań (No. 12/2005).

### Laboratory Analyses

The serum Fe concentration was determined with the colorimetric method by means of 2,4,6-tri(2-pyridylo)-5-triazine.

Diet and tissue samples for mineral analyses were digested with concentrated 65% spectra pure HNO_3_ (Merck) in a Microwave Digestion System (MARS-5, CEM, USA).

The concentration of copper (Cu), zinc (Zn), iron (Fe), magnesium (Mg) and calcium (Ca) in mineralised samples was determined with the flame atomic absorption spectrometry method F-AAS (Zeiss AAS-3, with BC, Germany), while the concentration of Cr was measured using a graphite furnace atomic absorption spectrometer GF-AAS (AAS EA 5, with BC, Jenoptic, Germany). The accuracy of Cu, Zn, Fe, Mg and Ca measurements was assured by simultaneous analysis of certified reference material (Pig Kidney BCR No. 186, Brussels), while the analysis of Cr was controlled using certified reference material (Virginia Tobacco Leaves CTA-VTL-2, Poland) (Table 2). The mean recoveries of certified levels (expressed as percentage of mean certified values) were as follows: Cu—103%, Zn—101%, Fe—97%, Mg—104%, Ca—103% and Cr—102%.

**Table 2:** The accuracy of the method of determination of elements (mean ± SD)

Element	Number of samples (*n*)	Certified value (μg· g^−1^)	Analytical value (μg· g^−1^)	Method accuracy [% certified value]
	Certified reference material Pig Kidney BCR No. 186			
Ca	6	295 ± 2	291.13 ± 7.46	98.7
Mg	6	830 ± 8	843.80 ± 11.25	101.7
Fe	6	299 ± 2	295.96 ± 11.53	99.0
Zn	6	128 ± 3	123.60 ± 1.52	96.6
Cu	6	31.9 ± 0.4	31.87 ± 0.20	99.9
	Virginia Tobacco Leaves CTA-VTL-2			
Cr	6	1.87 ± 0.16	1.81 ± 0.14	96.9

### Statistical Analyses

The data were presented as mean ± SEM. The results were analysed using one-way analysis of variance (ANOVA/MANOVA) and the Tukey’s test to determine significant differences (*p* < 0.05). All calculations were done using Statistica ver. 7.0 software (StatSoft, Tulsa, USA).

## Results

**Fig. 1:**
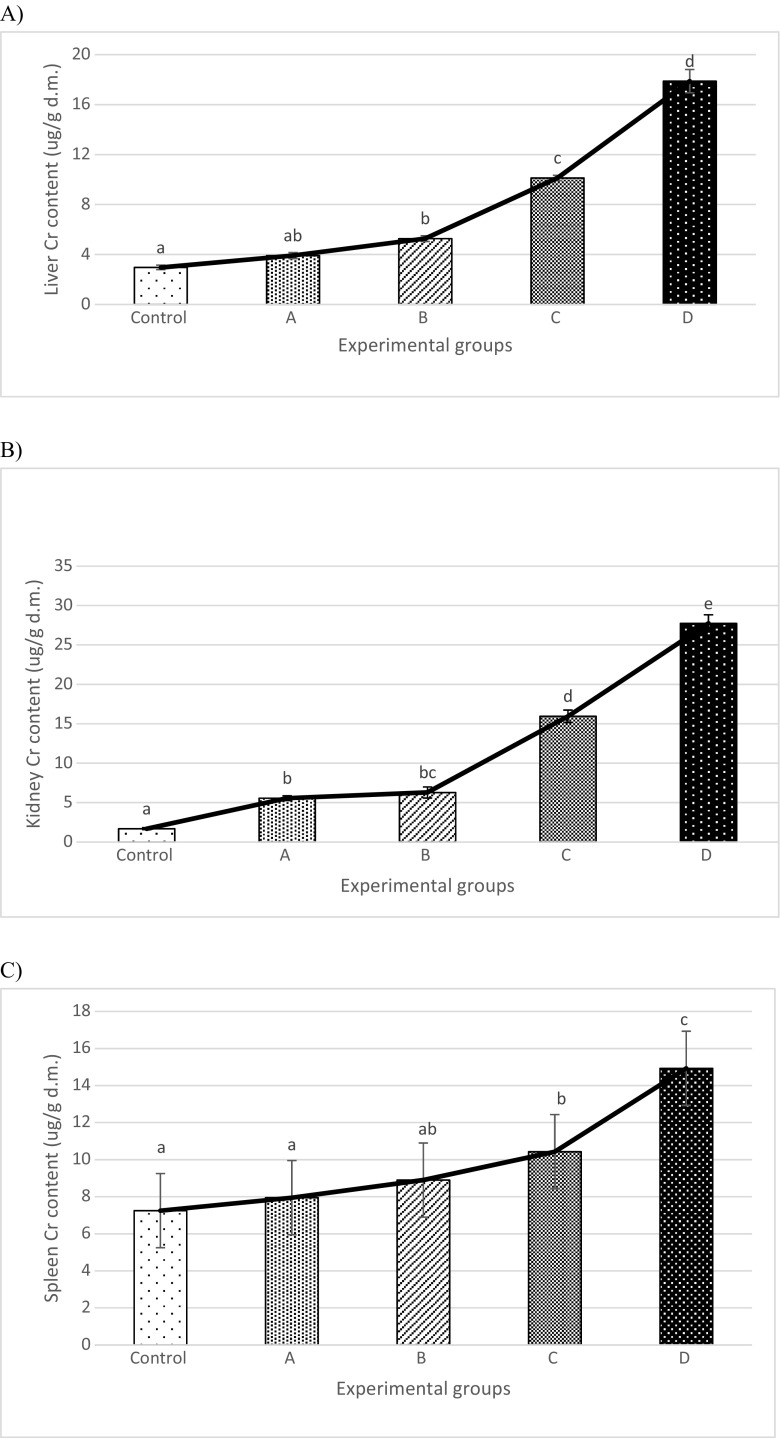
Chromium contents in tissues after Cr3 supplementation at doses 100, 200, 500 and 1000 mg Cr· kg^−1^ diet for 4 weeks in female Wistar rats, **a** liver, **b** kidney, **c** spleen, (microgram per gram dry mass). *Different letter superscripts* indicate statistically significant differences at *p* < 0.05. *Control* group, *A*—group supplemented with 100 mg Cr(III)· kg^−1^ diet, *B*—group supplemented with 200 mg Cr(III)· kg^−1^ diet, *C*—group supplemented with 500 mg Cr(III)· kg^−1^ diet and *D*—group supplemented with 1000 mg Cr(III)· kg^−1^ diet

Figure 1 and Table 3 show the effects of Cr3 supplementation on the tissular content of Cr, Cu, Zn, Fe, Mg and Ca in healthy female rats. As expected, supplementary Cr3 increased the liver and kidney Cr levels in a dose-dependent manner (Fig. 1a). The addition of Cr(III) to the diet at a dose of 100 mg of Cr· kg^−1^ did not significantly increase the liver Cr content (3.91 ± 0.24 vs. 2.96 ± 0.18 μg· g^−1^ dry mass). However, in comparison with the control group (2.96 ± 0.18 μg· g^−1^ d.m.), the doses of 200, 500 and 1000 mg of Cr(III)· kg^−1^ significantly increased the liver Cr level, by 78% (5.27 ± 0.23 μg· g^−1^ d.m.), 242% (10.13 ± 0.21 μg· g^−1^ d.m.) and 504% (17.88 ± 0.93 μg ·g^−1^ d.m.), respectively. Supplementary Cr3 at doses of 100–1000 mg of Cr· kg^−1^ increased the kidney Cr content by 229, 272, 844 and 1541%, respectively (Fig. 1b). All changes were statistically significant. Moreover, in the animals fed the diets containing 500 and 1000 mg Cr(III)· kg^−1^, spleen Cr levels were markedly higher, by 44 and 106%, respectively (Fig. 1c).

**Table 3:** The effect of supplementary Cr3 on the mineral status in healthy female rats (mean ± SEM)

Index	Experimental groups				
	Control (1 mg ·kg^−1^)	A (100 mg· kg^−1^)	B (200 mg· kg^−1^)	C (500 mg· kg^−1^)	D (1000 mg· kg^−1^)
	Cr status				
Liver Cr content (μg· g^−1^ d.m.)	2.96 ± 0.18^a^	3.91 ± 0.24^ab^	5.27 ± 0.23^b^	10.13 ± 0.21^c^	17.88 ± 0.93^d^
Kidney Cr content (μg· g^−1^ d.m.)	1.69 ± 0.12^a^	5.56 ± 0.31^b^	6.28 ± 0.71^bc^	15.95 ± 0.79^d^	27.74 ± 1.09^e^
Spleen Cr content (μg· g^−1^ d.m.)	7.25 ± 0.44^a^	7.95 ± 0.47^a^	8.90 ± 0.39^ab^	10.43 ± 0.47^b^	14.93 ± 0.79^c^
			Cu status		
Liver Cu content (μg· g^−1^ d.m.)	23.42 ± 0.70^a^	24.26 ± 0.66^ab^	27.15 ± 0.70^b^	24.22 ± 0.77^a^	24.04 ± 0.66^a^
Kidney Cu content (μg· g^−1^ d.m.)	57.38 ± 5.34	52.09 ± 2.08	55.55 ± 3.97	58.83 ± 2.79	49.30 ± 4.42
Spleen Cu content (μg· g^−1^ d.m.)	10.10 ± 0.70^ab^	13.57 ± 2.11^bc^	9.41 ± 0.42^a^	14.90 ± 0.52^c^	15.43 ± 0.57^c^
	Zn status				
Liver Zn content (μg· g^−1^ d.m.)	128.2 ± 5.2	122.3 ± 6.0	118.1 ± 3.5	129.7 ± 3.0	136.4 ± 6.0
Kidney Zn content (μg· g^−1^ d.m.)	110.1 ± 4.9^a^	120.2 ± 3.1^ab^	121.3 ± 6.8^abc^	141.7 ± 5.5^c^	132.8 ± 3.6^bc^
Spleen Zn content (μg· g^−1^ d.m.)	130.4 ± 4.0	118.7 ± 4.6	126.1 ± 3.8	122.0 ± 5.4	121.7 ± 8.5
	Fe status				
Serum Fe concentration (μg· dl^−1^)	296 ± 19^b^	248 ± 10^ab^	242 ± 15^ab^	246 ± 15^ab^	214 ± 15^a^
Liver Fe content (μg· g^−1^ d.m.)	1272 ± 42^b^	1202 ± 44^ab^	1163 ± 39^ab^	1002 ± 58^a^	1020 ± 54^a^
Kidney Fe content (μg· g^−1^ d.m.)	490 ± 21^c^	460 ± 20^bc^	429 ± 24^abc^	402 ± 19^ab^	375 ± 13^a^
Spleen Fe content (μg· g^−1^ d.m.)	7364 ± 554	7592 ± 484	7420 ± 438	7241 ± 354	8672 ± 615
	Mg status				
Liver Mg content (μg· g^−1^ d.m.)	851 ± 33	844 ± 37	801 ± 30	865 ± 25	868 ± 29
Kidney Mg content (μg· g^−1^ d.m.)	978 ± 28	980 ± 20	973 ± 18	1001 ± 42	979 ± 47
Spleen Mg content (μg· g^−1^ d.m.)	1078 ± 42	914 ± 22	964 ± 24	994 ± 32	994 ± 55
	Ca status				
Liver Ca content (μg· g^−1^ d.m.)	49.8 ± 1.4^c^	43.2 ± 1.6^c^	25.6 ± 3.1^b^	23.0 ± 1.0^ab^	15.7 ± 1.0^a^
Kidney Ca content (μg· g^−1^ d.m.)	226 ± 10	253 ± 14	264 ± 11	267 ± 11	267 ± 12
Spleen Ca content (μg· g^−1^ d.m.)	547 ± 52	548 ± 53	519 ± 51	486 ± 44	367 ± 38

The values in the same row that do not share the same superscript letter are significantly different (*P* < 0.05)

*Control* control group, *A* group supplemented with 100 mg Cr(III)· kg^−1^ diet, *B* group supplemented with 200 mg Cr(III)· kg^−1^ diet, *C* group supplemented with 500 mg Cr(III)· kg^−1^ diet, *D* group supplemented with 1000 mg Cr(III)· kg^−1^ diet, *d.m.* dry mass, *dl* decilitre

Supplementary Cr3 at doses of 100 to 1000 mg· Cr kg^−1^ had no effect on the kidney Cu content in the female rats. However, the dose of 200 mg Cr(III)· kg^−1^ significantly increased the liver Cu level by 16% (27.15 ± 0.70 μg· g^−1^ d.m.) as compared with the control rats (23.42 ± 0.70 μg· g^−1^ d.m.). High doses of Cr(III) (500 and 1000 mg· kg^−1^) increased the spleen Cu content by 48% (14.90 ± 0.52 μg· g^−1^ d.m.) and 53% (15.43 ± 0.57 μg· g^−1^ d.m.), respectively vs. control group (10.10 ± 0.70 μg· g^−1^ d.m.) (Table 3).

Supplementary Cr3 did not affect the liver and spleen Zn contents. However, in comparison with the control group (110.1 ± 4.9 μg· g^−1^ d.m.), the diets supplemented with 500 and 1000 mg Cr(III)· kg^−1^ significantly increased the kidney Zn level, by 29% (141.7 ± 5.5 μg· g^−1^ d.m.) and 21% (132.8 ± 3.6 μg· g^−1^ d.m.), respectively.

The effect of supplementary Cr3 on Fe metabolism was assessed on the basis of morphological and haematological blood indices, such as haemoglobin concentration (Hb), haematocrit ratio (HCT), the number of erythrocytes in the blood (RBC), mean platelet volume (MPV), mean corpuscular volume (MCV), mean corpuscular haemoglobin concentration (MCHC), red cell distribution width (RDW) (the data were presented in our previous paper) [27], serum Fe concentration and tissular Fe content (liver, kidney and spleen) (Table 3). The haematological indices under analysis in the rats supplemented with Cr3 were not significantly different from the control rats [27]. However, the highest dose of Cr 1000 mg kg^−1^ (100 mg· kg^−1^ b.m.) decreased the serum Fe concentration by 28%. As far as the tissular Fe levels are concerned, the Cr doses of 500 and 1000 mg· kg^−1^ significantly reduced the liver Fe content by 20% (1002 ± 58 μg· g^−1^ d.m.) and 21% (1020 ± 54 μg· g^−1^ d.m.), respectively, and the kidney Fe level by 18% (402 ± 19 μg· g^−1^ d.m.) and 24% (375 ± 13 μg· g^−1^ d.m.), respectively. No effects on the spleen Fe content were noted.

There were no significant differences in the tissular Mg levels of healthy female rats fed diets with Cr3 at doses of 100 to 1000 mg of Cr· kg^−1^.

The kidney and spleen Ca levels in the groups supplemented with Cr3 were not different than in the control group. However, supplementary Cr3 decreased the liver Ca level in a dose-dependent manner. The addition of Cr(III) at a dose of 100 mg· kg^−1^ to the diet had no effect. However, in comparison with the control group (49.8 ± 1.4 μg· g^−1^ d.m.), the doses of 200, 500 and 1000 mg· kg^−1^ significantly increased the liver Ca level, by 49, 59 and 68%, respectively.

## Discussion

Our previous studies showed that Cr3 was a relatively safe compound [18, 28, 29].

Publications concerning Cr are related to the transport, distribution and bioactivities of this element from various Cr compounds in different biological models.

The effects of the nutritional supplement Cr3 on healthy rats and rat models of insulin resistance and type 1 and 2 diabetes have been examined [4, 17, 27, 30–34]. Some reports have shown that chromium(III) has beneficial effects for the organism with disturbances of glucose and lipid metabolism [4, 17, 31]. However, data from the experiments conducted in healthy individuals quite often explicitly show lack of any favourable impact of Cr(III) on carbohydrates/lipid metabolism [27, 32–34]. Our study [27] showed no effect of Cr3 at doses 100–1000 mg of Cr(III)· kg^−1^ diet on serum glucose, total cholesterol, LDL-cholesterol, and HDL-cholesterol concentration (Table 4).

**Table 4:** The effect high dietary doses of Cr3 on blood glucose concentration and lipid profile indices in female rat (mean ± SEM) [27]

Index	Experimental groups				
	Control (1 mg· kg^−1^)	A (100 mg· kg^−1^)	B (200 mg· kg^−1^)	C (500 mg· kg^−1^)	D (1000 mg· kg^−1^)
Glucose concentration (mg dl^−1^)	105.7 ± 4.9	98.7 ± 8.6	98.8 ± 4.1	92.5 ± 2.5	94.8 ± 3.9
Total-cholesterol concentration (mg dl^−1^)	61.5 ± 8.4	72.2 ± 2.8	67.0 ± 5.5	63.3 ± 4.6	64.3 ± 2.6
LDL-cholesterol concentration (mg dl^−1^)	4.67 ± 0.49	5.17 ± 0.54	5.17 ± 0.54	5.00 ± 0.26	5.17 ± 0.87
HDL-cholesterol concentration (mg dl^−1^)	39.8 ± 3.6	41.5 ± 1.3	39.8 ± 1.8	37.2 ± 3.5	38.8 ± 1.0
TAG-triglycerides concentration (mg dl^−1^)	30.7 ± 1.9^ab^	32.2 ± 2.8^ab^	37.0 ± 3.0^b^	25.2 ± 2.4^a^	25.3 ± 2.6^a^

The values in the same row that do not share the same superscript letter are significantly different (*P* < 0.05)

*Control* control group, *A* group supplemented with 100 mg Cr(III)· kg^−1^ diet, *B* group supplemented with 200 mg Cr(III)· kg^−1^ diet, *C* group supplemented with 500 mg Cr(III)· kg^−1^ diet, *D* group supplemented with 1000 mg Cr(III)· kg^−1^ diet

Bennett et al. [34] found that Cr3 at doses of 1, 5 and 10 mg Cr· kg^−1^ lowered plasma insulin, leptin and triglycerides concentrations but had no effect on plasma HDL, LDL and total cholesterol after 10 weeks of treatment in male Sprague-Dawley rats. Healthy Sprague-Dawley rats treated daily with 20 μg· kg^−1^ body mass as Cr3 intravenously for 12 weeks had lower blood plasma insulin, total cholesterol, LDL, HDL and triglycerides but not glucose levels [31]. The data obtained by Herring et al. [32] strongly suggest that long-term (15-month) Cr3 supplementation does not significantly affect metabolic responses in blood glucose concentration to glucose and insulin in male Wistar rats consuming a normal diet or high-fat, high-carbohydrate cafeteria-style diet. Also Król et al. [33] confirmed that supplementary Cr3, given in the dosages 0.6 and 3 mg· kg^−1^ b.m. for 8 weeks, did not affect serum glucose, insulin and HOMA-IR index and serum lipid indices, except TAG (tended to decrease) in rats fed high-fat diet.

Unfortunately, the molecular mechanism by which chromium affects glucose and lipid metabolism is still unclear. This turn leads to the hypothesis that chromium ion supplementation have been beneficial only in disorders of glucose and lipid metabolism [35]. Most of the available literature exploring the effects of chromium supplementation in rats have been short-term studies [4, 25, 27–29, 36, 37]. Few have looked at the effect of long-term chromium supplementation [17, 31, 38, 39]. For the other roles of chromium in the body, we should use this supplement in a reasonable manner, being aware its possible side-effects.

However, there is little data on the influence of Cr on the mineral status in healthy individuals, particularly at pharmacological levels. Dietary supplementation with high doses of Cr(III) may disorder the status of other elements.

The accumulation of Cr in the liver and kidneys of rats receiving supplementary Cr3 was observed, but the results depended on the chemical form and dose of Cr. The Cr content in the liver, kidneys and spleen was found to increase in a manner dependent on the supply of Cr(III) in the diet. Other authors found similar relationships between the intake of Cr(III) and its concentration in the liver and kidney tissues in normal rats [40–42], in rats with diabetes mellitus type 1 and 2 [17, 30], in pig [43] and in quails [44].

Lindemann et al. [45] demonstrated differences between Cr sources in Cr concentrations in various tissues. In a study conducted by Yoshida et al. [37], the different effects of Cr on Cr concentration in the liver, kidney and femur were the result of the dietary Cr level (1, 10,100 μg· g^−1^ diet) than chemical form (CrPic vs. CrCl_3_). However, the greatest Cr concentration was found in kidneys, which was also confirmed in our study. Wang et al. [46] also reported that supplementary Cr at a dose of 200 μg· kg^−1^ from CrNano and CrPic for 40 days increased the blood Cr level, Cr content in the liver, kidneys and heart as well as faeces and urine in finishing pigs. Different results were obtained by Clodfelder et al. [4], who found that 24-week supplementation with Cr3 at doses ranging from 250 to 1000 μg Cr· kg^−1^ diet did not increase the content of Cr in the liver and kidneys in healthy rats or the animals with type 2 diabetes. It is assumed that the absorption and utilisation of Cr depend on its status in the gastrointestinal tract [46].

Some authors [47–49] indicated that prolonged supplementation with Cr(III) compounds may have negative impact on the metabolism of Fe due to the fact that Cr and Fe are bound with the same protein—transferrin. Human serum transferrin (Tf) is the iron transport protein responsible for delivering iron and a variety of other metals to cells [50]. This protein consists of two almost identical lobes, referred to as the C-lobe and the N-lobe, which can bind one metal ion each [51–53]. It was found that Fe^3+^ binding to the C-lobe is approximately 20 times stronger than Fe^3+^ binding to the N-lobe. Trivalent chromium (Cr^3+^) same for iron (Fe^3+^) typically binds to the C-lobe first, followed by loading into the N-lobe. Under normal conditions, only approximately 30% of the potential Fe^3+^ binding sites in Tf are occupied, leaving the unoccupied binding sites in either the C-lobe or the N-lobe, or both, to potentially bind other metal ions [51, 52]. Cr^3+^, Cu^2+^, Zn^2+^, Al^3+^, Ga^3+^, Ni^2+^ and Ti^4+^ are known to bind to Tf, and these metal ions could either compete with Fe^3+^ for Tf coordination or bind to the unoccupied lobes of Tf [54]. Cr^3+^ preferentially binds to the C-lobe of Tf, suggesting that it has the potential to compete with Fe^3+^ for that binding pocket. When saturation of transferrin with iron increases to over 50%, iron competes with chromium binding, affecting its transport [55]. The relationship between iron and chromium metabolism needs to be further investigated. It is not yet clear if chromium decreases iron absorption or if it is also involved in the downregulation of iron absorption [55]. It is also possible that exposure to high doses of Cr causes Cr^3+^ to bind to Tf and interferes with normal iron uptake, thus affecting Fe metabolism [1]. In case of oversupply of Cr, it may reduce Fe transportation to the cells, wherein the Cr-Fe interaction may occur already in the intestine, where these elements compete for a common site of absorption. A similar mechanism of interaction can also occur between supplementary Cr3 and Zn and Cu, as the absorption of these elements is interdependent.

In our study, the blood morphological indices remained unchanged, which indicates that Cr3 did not affect erythropoiesis [27]. A lower Fe level in the serum and tissue stores (in the liver and kidneys) was noted, but these changes occurred only after the application of very high doses of Cr(III) (50 and 100 mg Cr(III)· kg^−1^ b.m.). These results correspond to the study by Anderson et al. [47], who found a reduced Fe level in the tissues of rats supplemented with CrCl_3_. Also, Ani et al. [48] reported reduced transferrin saturation and tissue stores of Fe as well as lower haemoglobin and haematocrit index in rats fed a diet with a high dose of Cr(III).

Sun et al. [17] found that Cr3 administered to normal Sprague-Dawley (SD) rats at a dose of 20 μg· kg^−1^ b.m. for 24 weeks did not have a significant effect on the liver and kidney Fe contents. However, they observed higher liver Fe level and lower kidney Fe concentrations than their controls in Zucker obese (ZKO) rats [17]. Also, Love et al. [56] observed that the dietary Cr (16–2000 μg· kg^−1^ diet) given for 23 weeks had no effect on the blood iron level in Zucker lean (ZKL) rats. Similarly, Clodfelder et al. [4] did not observe adverse effects of Cr3 on the Fe status when given to healthy rats or to the animals with type 2 diabetes for 24 weeks as an aqueous solution at doses of 250–1000 μg of Cr· kg^−1^ b.m. In contrast, Cr3 supplementary in doses 10 and 50 mg· kg^−1^ diet for 8 weeks increased kidney Fe and spleen Cu contents but did not affect Zn status in rats fed with high-fat diet [33]. The studies by Shara et al. [38, 39] demonstrated that long-term supplementation with complex Cr(III) with niacin did not affect the metabolism of Fe in the rat (as assessed by the content of Fe in the serum, TIBC, RBC, haemoglobin and selected indicators of blood morphology). Prescha et al. [36] reported that supplementary Cr in a diet enriched with cellulose and/or pectin led to higher Cr and Fe contents in the femurs but did not change the Fe content in the liver, kidneys and muscles in male Buffalo rats.

Research on humans supplemented with Cr(III) at doses of 200–1000 μg· day^−1^ did not confirm significant effects on the Fe status. Campbell et al. [57] showed that 3-month supplementation of males aged 60–70 years with Cr(Pic)_3_ at an amount of 924 μg of Cr· day^−1^ had no effect on the Fe status. Similarly, Lukaski et al. [58] did not observe any changes in haemoglobin, haematocrit, serum Fe content, TIBC or transferrin saturation in 83 premenopausal women supplemented with Cr(Pic)_3_ at a dose of 200 μg Cr· day^−1^. Volpe et al. [59] found that 12-week supplementation with Cr(Pic)_3_ at a dose of 400 μg· day^−1^ combined with the exercise programme did not affect Fe or Zn levels in the serum of women with moderate obesity.

Anderson et al. [43] studied the effect of Cr(Pic)_3_ supplementation on the tissular levels of Fe, Zn and Cu in pigs. They found that Cr did not affect the level of these elements in the liver and heart but caused an increase in the Fe content and decrease in Zn and Cu contents in the kidneys.

Pechova et al. [60] observed that the addition of Cr(III) in the form of chromium yeast (5 mg· day^−1^ at the initial period of the experiment and increased to 8 mg· day^−1^ after 136 days) increased the Cu content, reduced the Mg and P levels in the serum, but had no effect on the plasma Zn concentration in young bulls.

Amatya et al. [61] conducted a study on broilers fed diets supplemented with 200 μg Cr· kg^−1^ in the form of CrCl_3_ and yeast chromium for 21 and 35 days. They found that the Cr level in the liver was lower than in the control group, the Cu content increased in the blood serum and liver, while the Fe and Mn content decreased in the liver.

Dębski et al. [62] reported an increase in the Cr and Cu content, but no change in the Zn level in the livers of hens fed a diet enriched with chromium yeast (0.5 mg· kg^−1^ dry mass) for 2 months.

Sahin et al. [44] showed that supplementary Cr(Pic)_3_ (200, 400, 800, 1200 μg Cr· kg^−1^) increased the Cr and Zn content but decreased the Cu level in the serum, liver, kidneys and muscles. The concentrations of Fe and Mg did not change in these tissues as dietary chromium supplementation was increased in the Japanese quail. Krejpcio et al. [63] found that supplementation with Cr3 (5 mg· kg^−1^ of diet) did not affect Mg levels in Wistar rats. Król et al. [64] showed that Cr3 supplementation disturb mineral homeostasis in the rats’ organs fed high-fructose diet. Cr3 increased Mg, Cu and Cr levels, although it did not influence tissular Ca, Fe and Zn contents, given for 4 weeks in doses 1 and 5 mg· kg^−1^ b.w. per day.

The addition of Cr(III) to the fibre-free diet and to the diets with cellulose or pectin did not change the Zn, Mg and P contents in the femur and Cr, Fe, and Zn levels in the muscles in rats [36]. However, the addition of pectin or cellulose to the diets, especially with Cr, increased the Zn content in the liver and kidneys and changed the Mg and Ca levels in these tissues [36].

Dogukan et al. [13] reported that supplementation with chromium histidinate (CrHis) increased the serum, liver, kidney Cr and Zn contents but decreased the Cu levels both in diabetic and non-diabetic rats. However, the serum, liver and kidney Fe concentrations were unchanged. In our previous studies [25], we observed that the Cu, Zn, Fe and Mg concentration in the liver of obese Zucker rats was lower than in ZDF and/or lean Zucker rats.

The role of Cr in the Ca of bone metabolism is unclear. CrPic_3_ has been found to reduce the urinary excretion of hydroxyproline and Ca in postmenopausal women, presumably indicating a reduced rate of bone resorption [65]. Evans et al. [65] observed reduced Ca excretion, an increased dehydroepiandrosterone level (DHEA) and reduced hydroxyproline to creatinine ratio in the urine of postmenopausal women after 60-day supplementation with Cr(Pic)_3_ at a dose of 200 μg· day^−1^, suggesting that Cr(III) could effectively prevent osteoporosis.

In other studies, the liver Ca content in lean rats was significantly increased by Cr3 and CrPic_3_ when administered at 1 mg Cr· kg^−1^ b.m., but the Mg level was not affected [25]. Prescha et al. [36] showed that the liver Ca content was very sensitive to supplemental Cr when given together with pectin and cellulose to Buffalo rats.

This study showed that high doses of supplementary Cr3 (100–1000 mg Cr(III)· kg^−1^ of diet) reduced the Ca content in the rat liver in a dose-dependent manner. The research by Sankaramanivela et al. [66] revealed that the exposure of male Wistar rats to K_2_Cr_2_O_7_ at a dose of 0.5 mg· kg^−1^ b.m. for 5 days increased the Ca content in the femur and cranial vault but reduced the activity of ALP and TRAP (resistant acid phosphatase tartrate), which the authors explained with low Ca resorption activity from the bone tissue.

The results of this experiment suggest that supplementary doses of Cr3 (100–1000 mg Cr· kg^−1^ diet; eq. ~10–100 mg Cr· kg^−1^ b.m^.^) given for 4 weeks did not affect the Mg status but influenced the Cr, Fe, Zn, Cu and Ca levels in healthy female Wistar rats.

In conclusion, high dietary Cr3 supplementation may affect the mineral balance in rat tissues.
